# Livestock Stakeholder Willingness to Embrace Preslaughter Stunning in Key Asian Countries

**DOI:** 10.3390/ani9050224

**Published:** 2019-05-08

**Authors:** Michelle Sinclair, Zulkifli Idrus, Georgette Leah Burns, Clive J. C. Phillips

**Affiliations:** 1 Centre for Animal Welfare and Ethics, School of Veterinary Sciences, The University of Queensland, Gatton, QLD 4343, Australia; c.phillips@uq.edu.au; 2 Institute of Tropical Agriculture and Food Security, Universiti Putra Malaysia, 43400 UPM Serdang, Selangor, Malaysia; zulidrus@upm.edu.my; 3 Environmental Futures Research Institute, School of Environment and Science, Griffith University, Brisbane, Nathan, QLD 4111, Australia; leah.burns@griffith.edu.au

**Keywords:** animal welfare, China, stunning, slaughter, livestock, Asia

## Abstract

**Simple Summary:**

Preslaughter stunning is a technical process by which animals are rendered unconscious prior to slaughter, as to avoid the pain and fear of being slaughtered. While it is a legislative requirement in some countries, it is not yet regularly practised in many countries. To better understand why this might be the case, this study conducted focus group sessions with leaders from the livestock industries in China, Vietnam, India, Thailand, Malaysia and Bangladesh. Leaders were asked to rate the willingness of livestock stakeholders to adopt preslaughter stunning, in addition to their suggested solutions for increasing the level of willingness, and their thoughts as to why they were or were not willing to adopt stunning. This data was analysed and presented within this manuscript. The findings were specific to each country, with similar themes shared across some of the countries. The findings of this study may aid in the development of programs that aim to increase the adoption of preslaughter stunning practices, with the purpose of improving animal welfare during slaughter.

**Abstract:**

Preslaughter stunning; the induction of unconsciousness and insensibility of animals prior to slaughter, is an important process for the welfare of livestock. The application of stunning is required by legislation in some countries, and rarely practised in others. In order to effectively advocate the implementation of stunning in the regions that do not include the practice as standard, it is first important to understand attitudes towards stunning, barriers to implementing stunning, and stakeholder willingness to embrace the practice. To this purpose, 17 focus group sessions were held with leaders in livestock production in China, Malaysia, Thailand, Vietnam, India and Bangladesh. Leaders were asked to rate their perceived willingness of livestock stakeholders to embrace stunning, and their rationales were discussed. In addition to this, the leaders were asked to present ideas to improve the willingness of stakeholders to embrace stunning. The data were qualitatively analysed used thematic analysis, quantified, and presented within this manuscript. Importantly, different attitudes and solutions existed by country, mostly in line with the predominating religion within the country, and the stage of economic development. Concerns around nonstatic and constantly evolving animal welfare benchmarks were also presented as important factors impacting the adoption of stunning, and the technical methods used. The findings of this study may aid in the development of programs that aim to increase the adoption of preslaughter stunning practices, to the purpose of improving animal welfare during slaughter.

## 1. Introduction

Stunning is defined as a technical preslaughter process subjected to individual animals to induce unconsciousness and insensibility, so that slaughter can be performed without avoidable fear, anxiety, pain, suffering or distress [1]. Stunning should be rapid (instantaneous in best practice), and should be sufficient to allow the animal to remain unconscious until the time of death [2]. 

Although originally developed as a method of immobilisation for ease of processing, stunning is now advocated primarily for animal welfare reasons, as a method of avoiding the stress of restraint for slaughter, the pain of the knife cut to the throat, and distress experienced during exsanguination [3,4,5,6].

Apart from exemptions for religious reasons, stunning of livestock before slaughter has been compulsory in the European Union since 1979, for the purpose of evading avoidable pain and suffering; the Federation of Veterinarians of Europe has the position that “the practice of slaughtering animals without prior stunning is unacceptable under any circumstances” [7]. While some European states, alongside countries such as Australia [8] and the United States of America [9] legislated obligatory stunning, they also make exceptions for slaughter that omits the practice for ritual, or religious, reasons. New Zealand, however, along with some states in Australia, has no exceptions to the requirement for stunning [10].

Reasons for not stunning are likely to differ between countries and regions, in the same way as attitudes to farm animal welfare [11]. However, concern about negative impacts on meat quality is a common documented reason for rejecting stunning [12,13]. While some of the science is conflicting about the validity of this concern, largely due to variation between methods and species, the scientific evidence suggests that meat quality is comparable between stunned and not stunned animals [4]. 

Another primary reason that stunning may not be widely practised in some areas of the world is connected to religious beliefs: stunning is not an accepted part of most ritualised slaughter, for example, Shechita slaughter for Kosher meat in Judaism, and most Halal slaughter in Islamism. However, collaborations between Islamic authorities and scientists in some countries are changing practices regarding the use of stunning in halal slaughter [3]. Recognising that under Islamic doctrine improving animal welfare is a godly duty and quoting doctrine such as “Whoever is kind to the creatures of God, is kind to himself” [14], Islamic scientists have been investigating alternative methods of stunning. Methods that do not cause death (before the animal dies from its throat being cut) or irreparable injury or damage to the animal prior to slaughter, for example, can be acceptable to halal authorities [3]. Other reasons for not stunning could include lack of knowledge about stunning or lack of access to appropriate tools and equipment. Poor stunning techniques can lead to a high level of stunning failure (an acceptable target is 5% of animals or less, according to Temple Grandin [15]. Problems include poor equipment, incorrect positioning, long hair on cattle, and delays in bleeding out [15].

The Food and Agriculture Organisation (FAO) considers stunning desirable, and notes that “most developed and many developing countries have legislation that requires preslaughter stunning” [16]; however, little is known about how widespread or consistently stunning is applied in some developing countries. This is despite the existence of guidelines under the World Animal Health Organisation that nearly all countries have adopted, which encourage, but do not require, stunning of livestock before slaughter [17]. 

A key region in this regard is Asia, where all of the world’s most important religions have large numbers of adherents, and which produces most of the world’s livestock, principally because this is where 57% of the population resides [18]. Practices in the different countries reflect religious beliefs, for example there is a requirement by the Malaysian Standards, ISO 1500:2009, for any animal that is stunned before slaughter to be alive post-stun and for the stun to not cause permanent physical injury, i.e., it must be reversible. China alone processes 39% of the world’s agricultural animals [18], and the region is home to over 57% of the world’s population [19]. The scale of agricultural operations, the number of animals that are slaughtered in the region, and the potential for pain and suffering at the time of slaughter suggest that better understanding of stunning practices in the region could provide significant benefit to animal welfare.

The purpose of this study is to investigate the willingness of livestock stakeholders in Asia to embrace stunning and to expose the barriers to adopting stunning practices. This information can then be utilised to develop targeted initiatives that address these issues in locally relevant and useful ways.

## 2. Method

This study was granted human ethics approval by the University of Queensland Ethics Committee, approval number: 2017000628. To gather data for this project, seventeen focus groups with a total of 139 participants were held in geographically dispersed locations across Vietnam (*n* = 20), Malaysia (*n* = 19), Thailand (*n* = 19), China (*n* = 23), India (*n* = 20) and Bangladesh (*n* = 43). Table 1 and Table 2 presents an industry segmentation of participants. Locations were chosen to be in geographically diverse regions of each country (e.g.; south, north, central, capital and regional) in an effort to capture potentially variable sentiments between domestic regions. Livestock industry leaders were invited to discuss the state of animal welfare in their country, in the context of major issues, challenges, solutions, opportunities and, as presented in this paper, perceived benefits to improving animal welfare. Participants were invited to attend the research sessions by country-based collaborators, and were selected based on criteria that they were leaders in the agricultural sector: that they represented private enterprise, domestic government (including government veterinarians attending the livestock industry), or agricultural academics, that they were currently employed in this industry, and that they had the ability to implement change into private businesses. The majority were private industry leaders (e.g., pig or poultry slaughterhouse or production managers or owners). Some participants were known to each other as professional colleagues.

Although plans were made for five to seven participants in each session, the actual number of participants present for each focus group varied from three to 13, as a result of last minute cancellations and increased interest, respectively. The mean length of the meetings was 3 hours and 45 minutes, with some extending past the scheduled 3.5 hours to enable all participants to contribute. 

Data was audio recorded during the sessions and additional written field notes were taken by a research assistant. The recordings and notes were collectively used to create abridged transcripts of each session. To avoid presenting potentially misleading data, linguistics and tone are not reported, as all data was translated, abbreviated, and summarised through a translator, from six different languages into English. Transcripts were uploaded into NVivo software for Mac 11.4.3 for analysis (QSR International, Melbourne, Australia).

To collect the data for this paper, participants were asked to rate the likelihood that livestock stakeholders would embrace stunning, as previously defined, on a scale from 1–10: 1 being that stakeholders would be extremely unlikely to adopt the practice and 10 being extremely likely. Participants were instructed that the rating given should be in the context of the necessary tools and equipment to stun the animals being available. After individually providing their rating verbally, they were then asked to give their reasons for these ratings. This was followed by the facilitator requesting further information for clarification where required. The participants were then asked to rate the opinions of the wider livestock community, rather than themselves personally, to avoid participants feeling defensive, and offering less honest results. The remainder of focus group discussion centred on specific animal welfare issues and solutions, and perceived benefits for improving animal welfare, which is reported elsewhere. 

### Analysis

Ratings of likelihood that stakeholders would embrace stunning were collated, and means, medians and modes are presented. Thematic analysis was then conducted on the data presented by participants pertaining to the justification of these rates, where persistent themes were identified and coded as nodes using coding software NVivo (QSR International, 2018, Melbourne, Australia). Due to the high level of diversity in justifications for not embracing stunning, data was then further analysed for frequent key words and themes, and presented alongside frequency scores. Data collected in response to ‘how to encourage stunning where it isn’t already used’ were also analysed for solution themes, and results quantified. Key quotes demonstrating the major themes were identified.

At the completion of the analysis, no new justifications emerged from the data, suggesting data saturation. The same lead researcher (MS) who conducted the focus groups also coded all themes and conducted the analysis. Particular attention was paid to careful analysis of the key themes (benefits), the frequency of their appearance between countries, the general context and meanings that had been applied to them by the participants, and how they related to one another. Word frequency functions were utilised to identify subthemes. Direct quotes are presented in the results according to the location in which they were collected (see abbreviations in Table 3).

## 3. Results

The following section presents tables that outline the ratings given by participants when asked ‘how likely are stakeholders within the industry to adopt preslaughter stunning’, followed by the key words identified with the highest frequency when participants justified the rating they gave. The second table outlines identified themes in the data when stakeholders were asked ‘how would we increase the willingness of stakeholders to adopt preslaughter stunning’. Given the varied nature of the solutions provided by participants in relation to that, quotes that best highlight the sentiment of each have been presented by country, and integrated into discussion on the existing agricultural landscape in that country, relevant to slaughter of animals.

### 3.1. Bangladesh

Animal agriculture in Bangladesh is mostly subsistence farming of goats, cattle and sheep [20]. Participants in the focus groups in Bangladesh reported that stunning was not widely practised in the country. The reason for this was religious in nature; ‘halal’, meaning ‘permissible’, in this instance, ‘permissible to eat’ [21] being the most common phrase, followed by ‘religious’ (Table 3). Specifically, they did not believe that meat resulting from animals that had been stunned before slaughter was halal. “I know there is no scope for stunning in halal, no way, no option” <DK>. According to their understanding and opinion, the primary reason that stunning was not halal was primarily due to the stunning restricting the subsequent exsanguination. “We believe that if we slaughter animal in halal way the animal will easily remove the blood” <DK>. “Preservation of the meat is better (without stunning), blood is good for bacteria if left inside meat, so it is also a food safety (concern)” <MY>.

Participants suggested that there were scientific studies that proved that not stunning the animal and following traditional halal slaughter methods were better for animal welfare: “we believe if we follow proper way of halal slaughter animal will feel less pain” <DK>. “This is not only the belief that this is the halal method, but we are researchers and educated people…we read comparative articles and studies and we saw and found that halal method is the less painful method for slaughter so far” <DK>. “We try to follow halal method not only better for religious but also scientific, a number have researched and found halal method is the best method” <SV>. However, details of these studies were not able to be recalled, and were sourced from “some social media and networks” <SV>.

Implemented during British rule, and not overturned or preceded, the Cruelty to Animals Act (1920) for Bangladesh states that a fine of two hundred Taka (approximately $2.30 USD) is deliverable for killing an animal in an ‘unnecessarily cruel manner’; however, it also states that ‘nothing in this section shall render it an offence to kill any animal in a manner required by the religion or religious rites’ [22].

When investigating potential solutions to increase the likelihood of the uptake of the stunning process preslaughter, it was suggested by participants that livestock stakeholders ‘need more information’. General knowledge of what stunning was, and how it is conducted, was limited amongst participants. “We can’t express our direct opinion right now as most people don’t have the knowledge, we must get clear about it” <SV>.

In four instances, antistunning positions were softened when participants were informed that stunning is being incorporated into the halal slaughter process in some other Muslim countries under scientific advice on acceptable methods for both animal welfare and halal, with the blessing of Islamic leaders. However, in another four instances, responses to that information resulted in statements about Bangladesh being different, with more fundamental interpretations of the doctrine. “Bangladesh people don’t eat snails like (they do in) other countries, Islamic following is stricter with slightly different beliefs” <DK>. “We are all are Muslims, but we are different” <DK>. In two instances, the question of likelihood to embrace stunning elicited animosity towards the facilitator; “Bangladesh is a Muslim country, our slaughter is better than all other slaughter methods, and we need justification first why you are asking about it…why do you want alternatives to our method?” <MY>. “Why is the stunning so important? We see a considerable welfare issue, as stunning can cause a lot of pain” <SV>.

This data suggests that any initiative to encourage uptake of stunning in Bangladesh should begin with education and training around the stunning process and the existing scientific research, and would be best to begin by engaging government and law makers. Initiatives led by Islamic authorities and engaging local trusted religious leaders are likely to be most effective (Table 4).

### 3.2. China

Responsible for 39% of the world production of agricultural animals [18], primarily pigs, chickens and fish (60% of the world’s fish), China has the potential to reduce suffering on a scale not offered elsewhere. A recent study into the perception of Chinese livestock stakeholders ranked ‘the absence of stunning at the time of slaughter’ as the most critical farm animal welfare concern in the country [23], indicating a potential opportunity to adopt stunning methods more readily in China. Chinese participants reported extremely high levels of willingness to embrace stunning, the highest in this study (Table 3). While many major production companies have adopted stunning methods (CO_2_ and electricity for pigs, and electric water baths for chickens), the practice is not common elsewhere. Religious beliefs did not play a significant role in the absence of stunning. This is reflected in previous studies, where religion was not a significant motivating force for Chinese livestock stakeholders [11], and corresponds with the fact that 77% of Chinese nationals consider themselves atheist, or not religious [24].

The concept of ‘quality’ was prominent in discussions about stunning in China (Table 3). “Based on the butchers experiences the stunning is harmful to meat quality” <GZ>. The meaning of quality was noticeably different in the South of China (Guangdong Provence) where concerns were presented, with some consensus, over the taste attributes being adversely affected in stunned meat, in addition to the overall meat quality, as compared to the participants in Mid and North China who focussed only on overall meat quality, and did not believe a taste difference existed. In the South it was reported that “stunning is harmful to the quality…it’s a different taste, (a different) flavour, not the special Chinese flavour” <GZ>. In the North, after consensus with all participants that stunning would not affect the taste, “stunning would be ok, doesn’t affect taste” <BJ>. Other key reasons for not undertaking stunning often centred around the lack of suitable equipment; “for me it’s the equipment (that) is hard to find sometimes” <BJ>, and further to that, the knowledge of how to use it in a way that is effective and doesn’t reduce meat quality; “Technical uncertainties also plays a part” <GZ>. “Better technique because at the moment the blood is not drained very well and leads (to) spots on the surface” <GZ>. Concern about stunning impeding the bleeding process was also prominent in each location in China. “It bleeds out totally, and bleeds quicker (without stunning)” <BJ>. To general agreement from the rest of the participant group in Zhengzhou, one participant stated “Without stunning the blood can get out more easily” <ZZ>.

When considering solutions to increasing the uptake of stunning in China, data in the study suggested an equal measure of concern about the appropriate tools and equipment being available, conducting technical training on how to use the tools and equipment appropriately, and to educating the increasingly discerning Chinese consumer (Table 4). Consumer education should address quality concerns, with a focus on taste in the south. Further research into the taste properties of stunned and not stunned meat have not previously been conducted, and may be useful. In line with previous studies, the implementation of animal welfare law would also be useful (none exists in China at the time of writing), as would the promotion of business benefits of stunning adoption amongst Chinese animal agriculture business owners.

### 3.3. India

As the second most populous country in the world [25], close to that of China, the scope of agriculture in India is also important in the world landscape. However, the nature and structure of Indian animal agriculture differs vastly from that of other countries. This is not only in the case of a reduced beef industry, on account of a majority Hindu population and beliefs in the sanctity of cattle, and the 30% of Indians who live a vegetarian lifestyle [26], but also because of the structure of Indian cast systems. As taking life is believed to result in bad Karma for the 80% of Indians who adhere to Hinduism [27], animal slaughter is commonly carried out by minority Muslim communities [28]. For this reason, in a country that is not populated with a majority Muslim population, beliefs around halal are highly relevant to the practice of stunning. This was supported by this study, with ‘awareness’, and ‘halal’ were the top relevant words (Table 3). While knowledge around stunning appeared low amongst participants in this study, they believed that stunning was rarely carried out in slaughterhouses in India, with the exception of those supplying the export markets. “One part of it is meat for export, and all those animals are stunned” <BL>. However, this was not able to be verified, and information about the slaughter and stunning practices in India are difficult to obtain. This may be in part to the closed nature of the communities tasked with coordinating slaughter for the population, and also due to the diverse and varied nature in which each state (and region) operates in India. In addition to this, scores around the perceived willingness of stakeholders to embrace stunning varied greatly between focus groups in India, and at times was contradictory. This may be indicative of the complexity of the Indian system, with India affectionately known as a land of contradictions [29]. This situation may be further complicated by the existence of large amounts of animal welfare legislation, that is largely not adhered to, and not logistically able to be monitored. The national ‘Prevention of Cruelty to Animals (Slaughter House) Rules, 2001’, stipulates that “every slaughter house as soon as possible shall provide a separate space for stunning of animals prior to slaughter”, additionally outlawing unlicensed slaughter men and slaughterhouses [30]. However, participants in each location during this study said that ‘legal’ slaughterhouses were rare, and in some situations, regions had a complete absence of legal slaughterhouses. “Bangalore is one of biggest cities in India…we don’t have a (legal) place to slaughter yet” <BL>.

Despite the law, slaughter continues, and remains unable to be monitored for logistical reasons. This may be the reason that, unlike in other countries in Asia [11], the presence of law is not a strong motivator in regards to animal welfare behaviours (Table 4), and also not presented as a key solution of justification in this study in regards to stunning specifically. It also may contribute to the varied and diverse nature in which animals are slaughtered in each region, and for vast differences in score regarding willingness to embrace stunning as presented in this study (Table 3).

The importance of considering community and livelihood in India was indicated with regard to stunning in this study, with both ‘community’ and ‘livelihood’ appearing in the most frequent words in this analysis (Table 3) see also [31].

The mention of animal sacrifice while discussing stunning was another area in which India was unique in this study. While stunning was believed to be an issue of religious consideration for Islamic communities when considering slaughter for consumption, the sacrifice of animals as a part of festivals, hosted mostly by factions of Hinduism, also do not involve stunning the animal. When asked if stunning could be included as a part of these sacrifices, it was stated “If the animal is stunned it will defeat concept of sacrifice”, with another participant clarifying that “If you make it not aware then you do away with sacrifice itself so stunning or not stunning is moot point” <KO>.

Large variations exist in the data across the regions in India. Therefore, we suggest addressing initiatives differently in each area regarding potential uptake of stunning practices. This should be initiated by conducting research to better understand the current practices in local areas, and tailoring a local action plan. 

One commonality, however, is the restriction of slaughtering to the Muslim communities and this represents an opportunity for a targeted approach. One such approach could be a demonstrative training and education program run by Muslim educators from local educational institutions that incorporates the requirements of halal with current scientific understanding, centred on the ability to successfully include stunning in halal slaughter. A program such as this should be monitored for success and ongoing investment. Scope also exists to promote both the religious benefits of stunning and improved animal welfare (such as an acquiescence to the need to consider animal welfare, as presented in the Islamic Hadiths) [32], alongside the business benefits of slaughter, particularly for those larger businesses in India.

### 3.4. Malaysia

Alongside Singapore and Brunei, Malaysia hosts the most developed economy in South East Asia [33], making the country an important agricultural leader for the region. Malaysia is an Islamic country, with close to 70% observance of Islam within the population, and Islamic Sharia Law constitutionally observed, adding to its agricultural importance of the country as a leading example in halal slaughter [34]. While stunning is deemed allowable (but not encouraged) by the governing Islamic body, JAKIM (Jabatan Kemajuan Islam Malaysia), and practised in some large poultry production companies because it makes the subsequent slaughter easier, complications exist around the appropriate usage of stunning. It is seldom used for species other than poultry. ‘Religious’, ‘halal’ and ‘JAKIM’ were some of the most common words Malaysian participants used when discussing stunning (Table 3), reflecting the perceived importance of ensuring meat is halal. However, the moderate approach to stunning is also represented by the moderately high ‘willingness to embrace’ rates given by participants in each location <7 out of 10 (Table 3). The general knowledge around what stunning entails also appeared much higher in Malaysia than in fellow Islamic majority country Bangladesh. 

The biggest concerns around stunning for halal also varied from that in Bangladesh, where the main concern was a potential impediment to complete bleeding. In Malaysia, the primary concern about fully embracing preslaughter stunning was one of direct interpretation of the halal protocol; ensuring that the animal is alive and not killed or irreversibly damaged at the moment of slaughter. “Most of them would support stunning, but they worry about death” <NS>. Methods of stunning that permanently damage the animal so it could not recover if it was not slaughtered would deem the meat defective and therefore not halal. “The general public are worried stunned animals may be dead … that’s the first thing, they need to be convinced that it doesn’t cause death” <NS>. 

The personal repercussions for leaders in livestock are high if they cannot guarantee that the meat they are feeding to trusting Muslim majority consumers is entirely halal. “If I do something wrong in halal certifying I will be held responsible even after life… no one wants to shoulder that responsibly, stakes are high” <NS>. Therefore, the motivation to ensure that products are certified halal is compelling. One of the major potential challenges presented within this study to animal welfare with was the incorrect usage of stunning equipment, particularly the wattage setting of electrical water baths for poultry. If the wattage is too high and the bird is killed, it is not halal and is therefore wasted. A wattage setting that is too low may result in immobilisation of the bird, but not unconsciousness, which presents animal welfare concerns. “They want to be sure the animal is not dead when (knife) cut…so they reduce the specifications so the animal is barely unconscious…one consultant from UK noted many of the birds after stunning were immobilised but not fully unconscious, it can cause more pain this way, but their biggest worry is the animal will die due to stunning” <NS>.

One solution includes the better training of workers on the production line checking the birds before slaughter. However, this presents logistical challenges. “They (the workers) are very well trained…but with the number of birds they can’t check them all” <NS>. Solutions suggested by participants included ensuring the stunning equipment was reliable and implementing random sampling to continually assess this reliability. 

Much trust was placed on JAKIM for religious oversight and on the Malaysian Government’s Department of Veterinary Services (DVS) for its delivery. “We (DVS) don’t have to show the public what we do, but they also do need to be sure and confident in what we are doing” <NS>. The continued close collaboration of both bodies on implementing stunning that meets animal welfare requirements and halal requirements is extremely important to the adoption of stunning in Malaysia. Animal welfare training by technical professionals such as DVS was also recommended to be offered to religious bodies who are accountable for drafting standards, such as JAKIM, to increase confidence that both animal welfare and halal requirements can be met.

The continued role of scientific research into stunning methods that reliably result in the recoverability of the animal also offers to provide methods that will increase adoption of stunning practices, particularly regarding the development of methods that reliably stun but do not kill the animal. The continued development and progressiveness of the scientific and religious interface in Malaysia could provide a useful example of the successful incorporation of stunning into certified halal practices in Muslim majority countries worldwide.

### 3.5. Thailand

As one of the most important chicken producers in the world, Thailand is host to a large export trade that aims to continue growing [35]. Along with neighbouring Vietnam, Thailand also has a focus on pork production. Participants in Thailand believed stunning to be routine in larger production enterprises, particularly for poultry, and less so in smaller to medium size slaughterhouses. They demonstrated a higher knowledge level of stunning than in the other countries, which was particularly the case in Bangkok where the head offices for the largest poultry producers are situated, and one of the focus groups were held. The consistent evolution of animal welfare benchmarks was cited as problematic by Thai participants, causing frustration and confusion, and representing a primary reason for not fully embracing stunning. This was particularly the case for large enterprises directing scientifically advised policy to improve animal welfare. “In the beginning say (the best method of chicken slaughter involves) electrical or gas stunning, now they say atmospheric so how will this end? They talk about control atmosphere for swine and we think maybe we will do that but a year later the research says ‘no’…once we implement a new method it costs millions and millions…then once we implement someone says ‘oh this is not welfare” <BK>. 

Findings from this study suggest that the confusion stakeholders may experience around the concept and technical details of stunning may be confounded by important international export partners who provide varied and sometimes conflicting advice. “We have experience buying a machine from France and they sent certificate saying (it) is approved by animal welfare (bodies)…then the British people come and say no…so we bought the machine and haven’t even used it, and we had to buy another one…then we get confused” <BK>. When the facilitator asked the participants if adhering to international guidelines such as the OIE (World Animal Health Organisation) Terrestrial Animal Code: Section 7 (Animal Welfare) would be useful, rather than attempting to cater to diverse national guidelines of importing countries, the answer was no. “For example in Thailand there is debate with OIE as OIE set up current rate for electrical stunning, then they asked Thai company to follow OIE standards… but we saw the chicken was dead and halal buyers said they would not accept it...so we informed OIE that the chicken is dead and other company inform them the same...industry has to collect all data for OIE but they don’t listen to us” <BK>. Universal approaches to stunning may not be applicable internationally due to the different breeds of animals; for example, Asian village chickens are smaller than standard European broiler chickens and are likely to be more susceptible to die as a result of electrical stunning. The lack of flexibility in importers to recognise research to adapt stunning for the maintenance of animal welfare under local conditions and with local breeds contributes to this problem; “if we have done the research and is suitable for Thailand but then export to Europe they say ‘no, you have to follow European standards’… many time we talk to them… we say how about a slightly different process but result is the same, they say not acceptable” <BK>. 

One solution to this challenge would be for OIE or international buying companies to implement addendum policy that allows for collaborative local research based on local conditions, that then allows for flexibility based on results, rather than a strict adherence to their own standards. This would allow results to be based on the best scientifically-measured animal welfare outcomes relevant to local conditions. 

A perceived benefit for embracing stunning for Thai participants, who are predominantly Buddhists, was an improved emotional state on viewing the animals’ death. “Thai people don’t like to see the suffering…people who work with animals don’t want to see the animals suffer” <BK>. 

For smaller companies outside of Bangkok, the main barrier to completely implementing stunning appeared to be the availability of tools and equipment, knowledge of how to use them, and the financial investment required. “If it’s a big company it’s no problem as they already have all the equipment but if small farmers they don’t have the stunning equipment” <CM>. The solution to address this, suggested by participants, was first to raise awareness about the business benefits of stunning. “Tell them the advantage of stunning but also need to tell them how to do it with less investment.” <KK>. Finally, it was suggested that the best way to implement stunning effectively in Thailand was not only to make the tools and equipment more available, but to ensure the equipment itself is intelligent. “The equipment itself must be intelligent so that it can change to different conditions by itself not by the workers…the person who should be influencing this is the scientists and engineers” <BK>.

### 3.6. Vietnam

Stunning is reported to be frequently adopted in large slaughterhouses in Vietnam that process imported animals, specifically for animals from countries like Australia that have implemented livestock welfare assurance schemes in response to public animal welfare interests [36]. However, the adoption of stunning practices outside these operations was believed by participants to be limited. One livestock leader from Ban Me Thout stated that “the animals all are treated badly in all size facility, including poor handling, and no stunning” <BT>, while another stated “currently many slaughterhouses do not apply stunning equipment” <BT>. A participant from Hanoi suggested that while larger slaughterhouses more often apply stunning, most of the small to medium operations do not: “Because they are different scales…intensive operations are happy to (apply stunning), but the small scale operations are different” <HA>.

The most discussed issue in Vietnam pertained to the availability of tools and resources, and knowledge of how to apply them for effective stunning was presented as the largest challenge, and the most important solution (Table 4) to increasing uptake of stunning practices in Vietnam. “They need the tools and resources but also need to look at training on how to use it” <HA>.

Some confusion existed around the methods of stunning that may be available, and in two of the three locations, participants stated that legislation in some provinces prohibits the possession of certain stunning implements, such as the penetrative bolt, due to it being considered a concealed weapon. “Some tools are not allowed to be used … the stun gun captive bolt is illegal in some areas, people think it is a weapon...they think it’s a gun only for military and police” <HC>.

Making the appropriate tools and resources available to the livestock industry as a solution was represented slightly more frequently than the potential impact of government involvement, legislation and monitoring (Table 4), and participants stressed the importance of coupling the availability of tools and resources with encouragement to use the equipment from a competent veterinary authority. “Some slaughterhouses are controlled by vet authority and have to provide (stunning) equipment…however many slaughterhouses are outside of the control of competent authorities” <BT>. Although this appears to be changing, “little by little all slaughterhouses are moving under control of vet authorities” <BT>.

Participants also suggested that if the slaughterhouse agrees to implement stunning, they should be supported to do so. “Agree very high/likely to use stunning tools but wonders about investment…no problem for big companies but for small scale I worry about the cost” <HC>. “If slaughterhouse owners agree and are willing, they should receive 50% of the resource support to buy the equipment” <HA>.

The second most discussed solution described the support of law, standards and monitoring. The ‘law’ was one of the most frequent words used when discussing stunning in Vietnam (Table 2), which is consistent with previous research that suggested the presence of a law would be the strongest motivator to improve animal welfare amongst Vietnamese livestock stakeholders [11]. “Most important is legal requirement/regulation…and strict enforcement; if there was a law it would be most the most important encouragement to embrace stunning” <HA>. Vietnam’s National Assembly passed an animal welfare law in November 2018 that makes preslaughter stunning compulsory [37,38]; however, formal details are not yet available, and when the law will be enacted is unknown.

Participants in the south of Vietnam reported the highest rate of willingness to embrace stunning, which could be influenced by the presence of large slaughterhouses in the region that cater to the aforementioned import industry. One participant in Ho Chi Minh City stated that he had reformed his business to include preslaughter stunning based on recent training and collaborations with exporting and industry bodies in Australia. “From our company perspective, some years ago I knew nothing about animal welfare, until I was trained with other people by an Australian company…then I became aware of the importance of animal welfare, I read more, got more knowledge and then I trained my staff. Our partner company initiated the training and we were trained by MLA (Meat and Livestock Australia)” <HC>.

The third most discussed solution to encouraging the uptake of stunning presented by Vietnamese participants was advocating the business benefits to stakeholders. This was believed to include “Saving labour costs, time, and money are the benefits of stunning and also better meat quality…better environment for animals, less noise…(there is) a direct benefit to people and workers…and meat quality” <HA>. However, this belief is not purported to be common amongst livestock stakeholders in Vietnam yet. “Most butchers say it’s not good because it effects the meat quality, but I know electric stunning is good, but not if applied in the wrong position” <HA>. When asked who might be best to advocate this, the veterinary authorities of Vietnam and the government Animal Health Department were again nominated. “I suggested a specific solution for Department of Animal Health to work with abattoirs and farms and butchers and meat processors and show that stunning is good for meat quality” <HA>. “Vet Authority because they already have experience working with abattoirs and show how to stun, and they work with them already; they inspect them” <HA>. “I think the authorities need to communicate with them; vet authorities… Department of Animal Health will be best to tell people it does not damage meat quality” <HC>.

### 3.7. Application across Cultures

When comparing the results across countries, it is more apparent that attitudes to preslaughter stunning fall under religious, meat quality, or resources contexts. The general context that is the most significant when relating to impediments to stunning varies by country, and is directly related to the religiosity of the general population that reside in the country. Where it is not a matter for meat quality and technical resources, the practice appears to hold ritualistic or spiritual relevance, likely given that the process results in death; the life phenomenon that religion most readily seeks to address and demystify for followers. Where religion or ritual is cited as the most important consideration in relation to the adoption of preslaughter stunning (Malaysia, Bangladesh, India), religious leaders could be usefully engaged in development initiatives. In countries where tools, resources and technical knowledge were a primary consideration (Vietnam, China and Thailand), solutions may be substantially simpler; and providing those elements are likely to result in a substantial increase of adoption. Where the impact on meat quality was an important factor; local collaborative investigations could be usefully conducted, to demonstrate the impacts or lack of impacts stunning may have on meat quality, and to move forward to address the situational factors that may contribute to a reduced quality, such as the incorrect application of stunning methods.

## 4. Limitations

Rates collected within focus group activity were not anonymous, and were shared freely, and so are susceptible to the impact of peer pressure and conformity. Qualitative data collected and analysed for themes was a small sample size relative to the industry they represent. In total there were 144 representatives across 17 locations, which is larger than any previous qualitative studies in this area. Further detailed studies to more rigorously assess the impact of the themes identified within this study, such as follow up surveys, are recommended.

## 5. Conclusions

This study identifies circumstances in which stunning could be adopted where it currently is not in a number of key countries in S, E and SE Asia. The reasons for adopting or not adopting preslaughter stunning differed across the six countries included in this study (and sometimes also between regions within countries, such as was the case in China). Potential solutions were suggested by participants that are relevant for livestock stakeholders interested in increasing the adoption of stunning practices for animal welfare reasons. These included the engagement of religious and scientific scholars to bridge gaps in understanding, to make available stunning tools and equipment, to provide technical training on the usage of these tools and resources, to advocate the business benefits for incorporating stunning into the process of slaughter, to engage government departments such as veterinary departments and animal health departments involved in inspections and policy, to raise consumer awareness and to continue scientific research. The emphasis of each of these solutions varied depending on the country of origin of the participants. An alignment of international rules and the development of capacity to approach locally encountered challenges as presented by Thai participants is recommended. While top-down legislative solutions are likely to be successful in countries such as China, it is important to note that the desired results are unlikely to be produced where laws are not able to be enforced, as demonstrated by Indian participants. Therefore, the need to tailor solutions by country and culture is fundamental.

The information presented in this study provides some insight into industry sentiments about the practice of preslaughter stunning, which could be used to better advise initiatives for increased uptake of the practice. 

A large number of animals are slaughtered for food in Asia and stunning is commonly agreed to be a very important welfare influence in this process. Therefore, informing successful initiatives to support uptake of preslaughter stunning is critical because it has the ability to reduce suffering on a large scale.

## Figures and Tables

**Table 1:** Participants by country.

Country	City/Town	Participant N	
**China**	Guangzhou	7	23
	Zhengzhou	7	
	Beijing	9	
**Vietnam**	Hanoi	7	20
	Ban Me Thout	5	
	Ho Chi Minh City	8	
**Thailand**	Bangkok	10	19
	Khon Kaen	3	
	Chiang Mai	6	
**Malaysia**	Negeri Sembilan	6	19
	Kuala Lumpur Selangor	13	
**India**	Banglaore	6	15
	Kolkata	5	
	Trivandrum	4	
**Bangladesh**	Dhaka	13	43
	Savar	13	
	Mymensingh	17	

**Table 2:** Breakdown of stakeholder participant roles within the livestock industry, by country.

Country	Stakeholder role			
	Private Industry Leaders	Private Industry Veterinarians	Government Representatives	Agricultural Academics
China	15	0	1	9
Vietnam	4	3	13	1
Thailand	11	4	2	2
Malaysia	9	5	5	1
India	3	5	1	6
Bangladesh	4	2	17	21

**Table 3:** Likelihood to adopt stunning, and key themes, by country and region (all stunning data).

Location (Country, City and Its Abbreviation)	N	Mean *	Keywords, in Declining Order of Frequency **
Bangladesh			Halal, religious, better, quality, different, awareness, equipment, knowledge, benefits, tools, rules, pain, Muslim, productivity, law, handling, blood
Dhaka <DH>	10	1	
Mymensingh <MY>	11	1	
Savar <SV>	10	1.8	
**China**			Quality, government, law, different, public, company, knowledge, benefits, tools, religious, already, equipment, consumers, methods, pigs, time, process, standards, chicken, improving, handling, best, butchers, implement, rating, accept, media, research, information
Beijing <BJ>	9	10	
Guangzhou <GZ>	6	9.8	
Zhengzhou <ZZ>	5	10	
**India**			Awareness, halal, government, religious, important, problem, issues, quality, improve, Muslims, butchers, food, livelihood, health, lack, community, equipment, show, aware, public, research, education, method, vet, accept, example, sacrifice, tools, chicken, handling
Kolkata <KO>	5	10	
Bangalore <BL>	6	1.1	
Trivandrum <TR>	3	1.3	
**Malaysia**			Religious, halal, meat, think, quality, know, important, DVS (Department of Veterinary Services, chicken, improve, JAKIM (Jabatan Kemajuan Islam Malaysia), public, industry, company, equipment, education, awareness, dead, process, benefits, workers, handling, money, time, Muslim, work, cost, method, education, butchers, knowledge
Kuala Lumpur <KL>	10	7.6	
Negeri Sembilan <NS>	5	7.8	
**Thailand**			Need, know, better, good, law, think, important, farmers, company, different, quality, halal, tools, already, want, chicken, religious, agree, improve, business, equipment, way, thinks, still, farm, local, workers, benefits, education, process, knowledge, training, feel, right, care, government, money, personal, handling, media, research, benefit, follow, production, times, educate, social, try, value
Bangkok <BK>	10	7.7	
Chiang Mai <CM>	6	10	
Khon Kaen <KK>	3	7.6	
**Vietnam**			Improve, law, meat, important, quality, better, need, halal, knowledge, equipment, think, training, tools, religious, know, vet, many, different, benefits, company, education, small, already, want, authorities, awareness, butchers, general, health, show, handling, less, owners, public, lack, problem, time, authority, max, min, provide, resources, staff, tell implement, method, new, used, control, food, Muslim, place, thinks, treatment, activity, work, chicken, children, difficult, disease
Hanoi <HA>	7	6.2	
Ban Me Thout <BM>	5	8.4	
Ho Chi Minh City <HC>	6	9	

* Mean and Median scores range from 1 (stakeholders are extremely unlikely to adopt stunning) to 10 stakeholder are extremely likely to adopt stunning). ** Keywords were presented for up to the 100 top words for each location. Connecting words (for example, ‘and’), conversational words, and obvious words (for example, ‘stunning’, ‘animals’) along with those not deemed relevant or enlightening to report by the researcher were not included. Note: Data was broken down into regions as some countries demonstrated significant regional variability (e.g., China).

**Table 4:** Key themes, by country, in response to the question: ‘How to encourage stunning where it isn’t being used?

Country	Total % of Total Solutions	% (*n*) of Solutions that Fit the Identified Theme						
		Religious Collaborations	Government Involvement, Legislation + Monitoring	The Availability of Suitable Stunning Tools and Equipment	Advocate Business Benefits	Technical Training Best Practice Stunning application	Public Awareness and Consumer Education	Scientific Research
**All countries**	100	26.08 (36)	22.46 (31)	13.76 (19)	13.04 (18)	10.14 (14)	7.97 (11)	6.52 (9)
**Bangladesh**	10.14	57.14 (8)	21.42 (3)	0 (0)	0 (0)	7.14 (1)	0 (0)	14.28 (2)
**China**	6.52	0 (0)	11.11 (1)	22.22 (2)	11.11 (1)	22.22 (2)	22.22 (2)	11.11 (1)
**India**	17.39	41.66 (10)	0 (0)	0 (0)	16.66 (4)	4.16 (1)	25 (6)	12.5 (3)
**Malaysia**	32.60	37.77 (17)	33.33 (15)	4.44 (2)	4.44 (2)	8.88 (4)	4.44 (2)	6.66 (3)
**Thailand**	9.42	0 (0)	23.07 (3)	30.76 (4)	38.46 (5)	7.69 (1)	0 (0)	0 (0)
**Vietnam**	23.91	3.03 (1)	27.27 (9)	33.33 (11)	18.18 (6)	15.15 (5)	3.03 (1)	0 (0)
